# Management of Patients with Vulvar Cancers: A Systematic Comparison of International Guidelines (NCCN–ASCO–ESGO–BGCS–IGCS–FIGO–French Guidelines–RCOG)

**DOI:** 10.3390/cancers17020186

**Published:** 2025-01-08

**Authors:** Stefano Restaino, Giulia Pellecchia, Martina Arcieri, Giorgio Bogani, Cristina Taliento, Pantaleo Greco, Lorenza Driul, Vito Chiantera, Rosa Pasqualina De Vincenzo, Giorgia Garganese, Francesco Sopracordevole, Violante Di Donato, Andrea Ciavattini, Paolo Scollo, Giovanni Scambia, Giuseppe Vizzielli, Gynecologic Oncology Group

**Affiliations:** 1 Clinic of Obstetrics and Gynecology, “Santa Maria della Misericordia” University Hospital, Azienda Sanitaria Universitaria Friuli Centrale, 33100 Udine, Italy; stefano.restaino@asufc.sanita.fvg.it (S.R.); pellecchia.giulia001@spes.uniud.it (G.P.); lorenza.driul@uniud.it (L.D.); giuseppe.vizzielli@uniud.it (G.V.); 2 PhD School in Biomedical Sciences, Gender Medicine, Child and Women Health, University of Sassari, 07100 Sassari, Italy; 3 Department of Medicine, University of Udine, 33100 Udine, Italy; 4 Gynaecological Oncology Unit, Fondazione IRCCS Istituto Nazionale dei Tumori di Milano, 20133 Milano, Italy; giorgio.bogani@istitutotumori.mi.it; 5 Department of Medical Sciences, Obstetrics and Gynecology Unit, University of Ferrara, 44121 Ferrara, Italy; cristina.taliento@unife.it (C.T.); grcptl@unife.it (P.G.); 6 Department of Health Promotion, Mother and Child Care, Internal Medicine and Medical Specialties (PROMISE), University of Palermo, 90127 Palermo, Italy; vito.chiantera@istitutotumori.na.it; 7 Unit of Gynecologic Oncology, National Cancer Institute, IRCCS, Fondazione “G. Pascale”, 80131 Naples, Italy; 8 Department of Women’s, Child and Public Health Sciences, UOC Gynecology Oncology, Fondazione Policlinico Universitario A. Gemelli IRCCS, 00168 Rome, Italy; rosapasqualina.devincenzo@policlinicogemelli.it (R.P.D.V.); giovanni.scambia@policlinicogemelli.it (G.S.); 9 Female External Genital Surgery Unit, Division of Gynecological Oncology, Department of Women’s, Children’s and Public Health Sciences, Fondazione Policlinico Universitario A. Gemelli IRCCS, 00168 Rome, Italy; giorgia.garganese@policlinicogemelli.it; 10 Gynecologic Oncology Unit, Centro di Riferimento Oncologico, National Cancer Institute, 33081 Aviano, Italy; fsopracordevole@cro.it; 11 Department of Gynecological, Obstetrical and Urological Sciences, “Sapienza” University of Rome, 00185 Rome, Italy; violante.didonato@uniroma1.it; 12 Woman’s Health Sciences Department, Gynecologic Section, Polytechnic University of Marche, 60121 Ancona, Italy; a.ciavattini@staff.univpm.it; 13 Department of Obstetrics and Gynecology, Cannizzaro Hospital, University of Enna “Kore” (D’Agate and Scibilia), 95126 Catania, Italy; pscollo@unict.it

**Keywords:** vulvar cancer, guidelines, disease management, review literature

## Abstract

Background: Vulvar carcinoma is an uncommon gynecological tumor primarily affecting older women. Its treatment significantly impacts the quality of life and, not least, aesthetics because of the mutilating surgery it requires. Objectives: The management requires a multidisciplinary team of specialists who know how to care for the patient in her entirety, not neglecting psychological aspects and reconstructive surgery. How do the guidelines address multidisciplinarity, team surgical management, passing through preoperative diagnosis, and follow-up in such a challenging rare tumor to treat? Methods: To answer these questions, we compared the main scientific recommendations to identify similarities and differences in diagnostic and therapeutic management to provide an overview of the gaps that there are currently in European and American international recommendations in providing management guidance in a cancer that is both among the rarest and most difficult to manage. In this way, we aim to encourage an update in practices based on the latest scientific evidence. Results: A review of various international guidelines, some dating back to 2014, shows significant variation in approaches, ranging from initial diagnostic procedures to managing relapses. The most recent guidelines also lacked references to the latest literature, indicating that more robust scientific evidence is needed before new treatments, such as electrochemotherapy for palliation and reconstructive surgery post exenteration, can be widely adopted. Conclusions: From the systematic comparison of the main international guidelines, a strong heterogeneity emerged in the diagnostic and therapeutic recommendations as well as for the multidisciplinary approach that today is essential. Our work certainly stimulated an update of the main guidelines.

## Simple Summary

Vulvar tumors are uncommon and have a considerable impact on the functional and aesthetic well-being of those affected. Their treatment necessitates a comprehensive, multidisciplinary approach at various levels, highlighting the importance of having standardized recommendations that are aligned with the latest scientific findings. Are scientific guidelines aligning with the advancements made in this field of oncology, spanning from diagnosis to palliative care at various levels? To address this, we conducted a systematic comparison of the main European and American guidelines for vulvar cancer management to assess their current status of update. From our comparisons, many divergences emerged in management strategies. Among them, lack of reference to the most up-to-date diagnostic classification systems, indication for an integrated gyneco-oncologic and plastic surgical approach to postoperative management with the most modern advanced dressing devices and palliative setting with the use of immuno- and electrochemotherapy.

## 1. Introduction

Vulvar cancer (VC) constitutes 5% to 8% of gynecologic malignancies, with a median diagnosis age of 68 years. Although epidemiological data on the incidence and mortality in developing countries are limited, information from the International Agency for Research on Cancer (IARC) indicates that in 2019, VC mortality was highest in Europe (41.1%), followed by Asia (31.6%), North America (10.1%), Latin America, and the Caribbean (8.3%), Africa (8%), and Oceania (0.8%) [1].

According to data from the SEER database, the 5-year survival rates are 85.6% for localized disease (stages I/II), 47.5% for regional or locally advanced disease (stages III/IVA), and 23.3% for stage IVB patients (including those with pelvic nodal disease), according to the 2018 FIGO staging system [2].

Squamous cell carcinoma (SCC) is the most common malignant vulvar tumor. Two-thirds of them arise via a human papillomavirus (HPV) independent pathway with a more aggressive behavior than HPV-associated ones [3].

Most vulvar malignancies are diagnosed at early stages because they develop gradually and typically stay confined to one area for an extended period, progressing into invasive cancer over approximately 8 years [4] and are located in the labia majora [5].

The prevalence of HPV-related VC differs depending on geographic location, with HPV-dependent ones being most common in low- and middle-income countries [6]. Changing sexual behaviors and increasing HPV exposure, however, made the incidence of VC increase notably in high-income countries, particularly among women under 60 [7].

This rise is due to various factors, that are different for HPV-related vs. HPV-independent VC such as increased exposure to HPV, tobacco use, autoimmune disorders, and HIV coinfection. For HPV-independent VC, other contributing risk factors include vulvar lichen sclerosus, chronic inflammation, and the role of metabolic syndrome is debatable.

Vulvar cancer is often diagnosed later than its onset either for social reasons, especially in older women or due to a lack of timely recognition. Symptoms are usually long-lasting itching (a feature in common with LS and LP other than benign atrophy), burning, bleeding, and atypical genital discharge, and may sometimes manifest as raised nodules or ulcered lesions.

HPV vaccination strategies are expected to help prevent HPV-related vulvar neoplasia, with studies showing decreased precancerous lesions [7,8]. Prophylactic vaccination against oncogenic HPV types 16 and 18 has already led to a significant reduction in the development of preinvasive HPV-related vulvar lesions [9]. The impact this will have on the reduced incidence of associated cancers can be assessed in the coming decades.

Block-type immunoreactivity for p16 represents a surrogate for the diagnosis of HPV-dependent SCCs. It has become available in most laboratories and should be reported on the histological examination.

HPV-related vulvar precancerous lesions, typically high-grade intraepithelial lesions (vulvar HSIL), often arise on the labia and other external genital areas, with smoking and immunosuppression as significant risk factors; vaccination is expected to reduce their prevalence [10]. In contrast, HPV-independent vulvar intraepithelial neoplasia (VIN) primarily affects postmenopausal women with chronic inflammatory dermatological diseases: an increased risk has been shown for lichen sclerosus (LS) and lichen planus (LP) [11]. It often presents with pruritus, burn, and pain, with p53 mutations linked to the poorest clinical outcomes [5].

The entity known as VC not only encompasses two distinct etiopathogenetic pathways, but it comprises various diseases with unique precursors, leading to different histological subtypes. These range from HPV-related p16+/p53wt warty carcinoma, to keratinizing, HPV-independent p16-/p53-mutated carcinoma, and intermediate p16-/p53wt forms, which may progress to keratinizing or verruciform carcinoma. These subtypes have differing prognoses and potentially distinct treatment responses, which are still under investigation.

Multidisciplinary management begins in the diagnostic phase. Dedicated vulvoscopy clinics play a crucial role in identifying suspicious areas that require biopsy during the follow-up of chronic anogenital conditions.

Surgical management, as recommended by major scientific societies, necessitates centralizing patients who are candidates for these extensive surgeries in referral centers. These frameworks ensure adequate care and the development of long-term postoperative plans, which are crucial given the high rate of postoperative complications associated with surgeries requiring specialized clinical expertise. In terms of surgical staging, discrepancies between the 8th edition of the AJCC TNM staging system (2018) and the updated 2nd FIGO staging (2021) make direct correspondence challenging. Specifically, AJCC stage IIIA aligns with FIGO stage IVA when there is extension to the upper two-thirds of the urethra or vagina, or involvement of the bladder or rectal mucosa [12,13]. Furthermore, FIGO 2021 included a new way of measuring depth of invasion, by which a portion of the former FIGO stage IB patients will now be classified as FIGO stage IA [13].

Scientific research in VC is also progressing, with advancements not only in the surgical field—with the unavoidable novelties in plastic surgery reconstruction—but also in immunotherapy, as evidenced by recent publications for this tumor [14,15,16,17,18].

Equally significant is the advancement in electrochemotherapy, a palliative treatment option aimed at alleviating symptoms related to the bulk and pain of the neoplasm, particularly in elderly patients or those ineligible for other treatments [19,20,21].

Documented results from expert oncology groups show its usefulness and effectiveness, even for repeated sessions over time [12].

How do the different recommendations deal with these above-mentioned aspects?

Public health programs for early diagnosis of VC and the training of specialists for its treatment need to be sufficiently developed, in light of the recent epidemiological data.

Precisely because VC is a rare tumor, its management is challenging due to the complex health conditions of affected patients, the specialized surgical skills required, and the frequent long distances between reference centers and patients’ homes, necessitating remote treatment options. For all these reasons, it was necessary to summarize the latest recommendations from leading scientific societies on this topic, aiming to encourage timely updates that consider imperative a comprehensive, multidisciplinary, and ultra-specialist approach.

Building on these considerations, we wondered what the latest European and American guidelines on the topic were in order to stimulate any relevant updates and to provide clinicians facing their management in daily clinical practice with an easily accessible tool with the main recommendations summarized.

## 2. Materials and Methods

A systematic search of the latest published American and European guidelines regarding vulvar cancer’s treatment was conducted on PubMed and official websites of the leading international scientific societies. The search was performed from the beginning until today by selecting the latest version of each of them. We have excluded recommendations based on pre-2009 FIGO staging, considering it is too dated compared to more recent staging systems updates [22].

The following were indeed the most recent versions by the leading international scientific gynecological oncology’s societies selected for comparison in the review:

– National Comprehensive Cancer Network (NCCN) 2024 [5];
– American Society for Clinical Oncology (ASCO) 2024 [23];
– European Society of Gynaecological Oncology (ESGO) guidelines (updated 2023) [24];
– British Gynaecological Cancer Society (BGCS) 2023 [25];
– International Gynecologic Cancer Society (IGCS) consensus meeting 2022 for treatment of VCs in limited resources settings [26];
– International Federation of Gynaecology and Obstetrics (FIGO) cancer report 2021 [14];
– French guidelines 2019 from “Les recommandations de l’Assistance Publique—Hôpitaux de Paris” [27];
– Japan Society of Gynecologic Oncology (JSGO) 2015 [28];
– Royal College of Obstetricians and Gynaecologists (RCOG) 2014 [29]. The latter include indications on reconstructive surgery after radical surgeries up to exenteration that are present in only a few other recommendations.

The IGCS 2022 drafted a fascinating document summarizing recommendations for the management of this malignancy in social contexts where access to treatment is limited and disease incidence higher, especially in its forms related to HPV infection, because of poor vaccine uptake. However, it must be considered that invasive HPV forms account for about 30% of the total, and that at least another 20 years will pass before vaccinated cohorts reach the age of invasive vulvar carcinoma. We have included the recommendations therein for further sub-comparison [26]. All included recommendations were representative of the major players in the gyneco-oncology landscape internationally and easy for readers to access. All manuscripts were subjected to careful reading and analysis by three experts in the field (S.R., G.P., and M.A.), and any discrepancies in interpretation and comparison were resolved by discussion with the other authors. Since the different guidelines refer to other stadia, e.g., FIGO 2021 to the new one of the same name and ESGO to the AJCC TNM 8th edition, we will cite each staging system as it applies to the specific recommendations. Specifically, IGCS and ESGO recommends the AJCC staging system; ESGO specifies that, due to significant differences, AJCC and FIGO stages are not interchangeable for guiding therapy and stage-specific recommendations. ASCO and JSGO recognize both staging systems. On the other hand, NCCN, BGCS, and FIGO all use FIGO 2021, while the French, Japanese, and RCOG guidelines continue to use FIGO 2009 because they were written before the publication of the new FIGO staging system, so requiring an update. The treatment of vulvar melanomas and other rare vulvar tumors, addressed only in the NCCN, FIGO, BGCS, JSGO, and RCOG guidelines, was excluded from this comparison as it is not covered in all guidelines. Therefore, we will present our findings based on a comparison of selected recommendations related to diagnostic work-up, early and advanced stages treatment, follow-up, and relapse treatment.

## 3. Results

### 3.1. Diagnostic Work-Up

IGCS and JSGO do not include recommendations for diagnostic work-ups.

#### 3.1.1. Clinical Examination

Initial clinical examination must include precise documentation of the lesion’s anatomical extent (such as involvement of the labia minora and/or labia majora, clitoris, urethra, anus, perineum), its laterality and focality (unifocal/multifocal), as these details are crucial for pathological assessment. In this regard, ESGO, BGCS, and RCOG all recommend taking pretreatment photographs to aid in treatment planning, with BGCS specifically advising photos before and after the diagnostic biopsy. For FIGO, they must be taken by the pathologist on the excised piece. All other authors make no mention of it.

RCOG states that in certain situations where the clinical diagnosis is precise, and the patient is experiencing severe symptoms, such as heavy bleeding or pain, biopsy can be omitted and radical surgery on the vulvar lesion may be directly scheduled. However, it is recommended to perform a biopsy with a frozen section before proceeding with any radical procedure [29]. Furthermore, RCOG, which aligns with the NICE recommendations, adds that for large tumors, palpation should be conducted to determine if the cancer is infiltrating deep into the pubic and ischial bones. This examination may need to be performed under general anesthesia due to the pain often associated with these situations, including evaluation for groin lymphadenopathy [29].

A complete inspection of the vagina and cervix with cervical cytology for detection of HPV-related lesions is considered part of the diagnostic work-up by NCCN, ASCO, ESGO, and French guidelines, except RCOG and BGCS, which confines the indication to women with undifferentiated vulvar intraephitelial neoplasia in the pre-diagnostic phase of overt malignancy. ASCO also includes possible indications for oropharyngeal cytology. French guidelines add that in case of an abnormal cervico-uterine smear, a cervical examination should be followed by colposcopy [27].

#### 3.1.2. Biopsy

The guidelines unanimously agree that diagnosis should be made by performing biopsies on all suspected vulvar lesions, with careful mapping, orientation, and detailed descriptions of each lesion. However, they do not all provide the exact recommendations on the type of biopsy to be performed. In particular, two different currents of thought in general are recognized: an incisional and an excisional biopsy. In the first case: this is a biopsy sampling aimed at obtaining a suspicious tissue fragment for histologic analysis but without guaranteeing complete removal of the neoformation. In the second case, on the other hand, the lesion is completely excised with dual diagnostic and therapeutic purposes. Certainly, incisional biopsy is an option to consider in the case of large lesions, when the suspected lesion does not have clear resection margins so biopsy is used to define its nature to subsequently plan optimal surgical resection, in the case of advanced and metastatic disease, or, which is very common in vulvar cancers, if radical biopsy does not allow preservation of organ function.

Conversely, an excisional biopsy is preferred in cases of small, well-demarcated lesions, with a high likelihood of cancer and where the lesion is resectable with clear margins, and in early-stage cancers where it also serves as a treatment, for anatomo-pathological evaluation of the margins in order to define whether additional surgery or treatment is required. In vulvar tumors, incisional biopsy is diagnostic; excisional biopsy is both diagnostic and therapeutic. Therefore, in summary, tissue conservation, surgical planning, and impact on treatment decisions guide clinical rationale; while incisional biopsy is useful for diagnosis in more complex or advanced cases, excisional biopsy is often preferred for smaller, well-defined lesions or when immediate therapeutic resection is feasible. ESGO, BGCS, FIGO, French, and RCOG guidelines specifically recommend using incisional or punch biopsies and advise against excisional biopsies. Excisional biopsy should be avoided for the initial diagnosis, as it may complicate subsequent treatment planning [24]. BGCS asserts that an incisional biopsy (either punch or wedge) should ideally include the edge of the lesion, where there is a transition from normal tissue to abnormal tissue. It is important to avoid taking biopsies from a central ulcer, as this may not provide a diagnostic sample. The biopsy should also be of sufficient depth to distinguish between superficially invasive lesions and those with invasion greater than 1 mm, as this distinction is crucial for guiding further management [25].

American and French recommendations instead refer generically to a diagnostic biopsy [5,23,27].

#### 3.1.3. Imaging

According to ESGO guidelines, no imaging is needed for pT1a tumors, but ultrasound of inguinofemoral lymph nodes (IFLN) is recommended for those eligible for sentinel node biopsy (SLN), being accurate in predicting non-palpable metastases [30]. For other cases, systemic staging with computed tomography (CT) scans or 18F-FDG-PET/CT is advised, and if suspicious nodes are found, a biopsy is recommended if it impacts treatment [18]. For invasive tumors (≥T2) or unclear findings, magnetic resonance imaging (MRI) is recommended to assess extra-vulvar structures, while specialized centers may use transvaginal, transrectal, or perineal ultrasound for local staging. The ESGO authors recommend following the 2021 Vulvar International Tumor Analysis (VITA) guidelines for ultrasound features of affected metastatic vulvar cancer lymph nodes and the 2021 European Society of Urogenital Radiology (ESUR) MRI guidelines [31,32,33,34]. The VITA criteria, developed by the Vulvar International Tumor Analysis (VITA) group, provide standardized terms, definitions, and measurements for the sonographic evaluation of lymph nodes, particularly in the context of vulvar cancer. These criteria aim to ensure consistent and accurate ultrasound assessments of lymph nodes, with a focus on identifying features that may indicate metastasis or malignancy. Lymph-node features to be assessed are: size (maximum short axis diameter); shape (evaluating the roundness); echogenicity (assessing cortical thickness and changes in echogenicity); hilum (presence of fatty hilum, if loss, this is a sign of malignancy); margins (smooth or irregular), vascularity (patterns of blood flow on Color Doppler—peripheral or disorganized is a malignant sign).

VITA group consensus opinion, apart from ESGO, is mentioned only by IGCS recommendations for limited resources settings where they consider ultrasound is readily available and is more cost-effective compared to other imaging methods for VC staging [31].

All other documents from scientific societies published after 2021 do not reference ultrasound as a preoperative staging method.

NCCN guidelines exclude ultrasound from basal diagnostic work-up and list the remaining imaging options, such as CT, PET/CT, and MRI, for staging purposes, without specifying their site-specific intended purpose. Additionally, NCCN recommends examination under anesthesia (EUA) when appropriate and HIV testing for younger patients [5].

ASCO’s diagnostic work-up includes gynecologic examination, screening for other HPV-associated tumors, HIV testing, MRI, chest–abdomen CT, and 18-FDG PET/CT for positive nodes. Ultrasound (US)-guided fine needle aspiration is recommended for T3/T4 stages with enlarged inguinal nodes [23].

BGCS, like ESGO, does not recommend PET-CT for routine staging of vulvar cancers due to its limited accuracy in detecting small or necrotic lymph node metastases and the potential for false positives in inflammatory nodes. While ultrasound has shown better sensitivity and specificity, its effectiveness is operator-dependent. BGCS does not reference the European VITA consensus [31].

BGCS and French guidelines recommend clinical examination and radiologic staging to rule out gross nodal involvement, which is considered standard of care. If an SLN biopsy is planned, imaging of the groin—via ultrasound, MRI, or CT—is crucial to detect potential lymph node metastases [13,25,27].

They still recommend using fine needle aspiration (FNA) or core biopsy to assess suspicious lymph nodes when the findings could influence primary treatment, such as in SLN biopsy.

Further staging with CT/PET-CT is recommended in the presence of confirmed metastatic disease (i.e., positive lymph nodes) and/or in advanced disease before radical surgery [25].

In contrast, FIGO states PET-CT is more effective than CT in assessing and detecting IFLN involvement. This capability influences the planning of primary surgery and inguinal lymph node dissection, potentially allowing for optimal surgical extent without needing sentinel lymph node dissection or frozen sections [13,35,36].

ESGO authors [18] highlight the importance of onco-geriatric assessment, also recommended by ASCO and French guidelines [23,27].

A summary of further diagnostic comparison results is provided in Table 1.

### 3.2. Prognostic Factors

Among the various scientific recommendations, there is no clear division between prognostic factors of recurrence and survival. On the evaluation of margins, a new consideration is on the pathological final examination of surgical margins.

The depth of invasion (DOI) refers to the vertical measurement from the epithelial–stromal junction of the adjacent normal tissue to the deepest point of tumor invasion according to the FIGO staging system for vulvar cancer [13].

According to NCCN 2024, factors that may predict recurrence and/or survival include DOI, pathologic margin distance, tumor thickness, and lymphovascular space invasion (LVSI). LVSI is considered an adverse prognostic factor in vulvar squamous cell carcinoma by ESGO, though its prognostic significance remains not fully understood. ASCOs points out that surgical margin involvement is the most important prognostic factor [23]. According to BGCS, DOI is an independent prognostic factor that, along with tumor size, helps to differentiate between FIGO stage IA and stage IB tumors [25].

The revised 2021 staging system incorporates imaging for stage assignment. For stage I disease, DOI is now measured from the basement membrane of the nearest dysplastic rete peg to the deepest invasion point. Stage IIIA now includes involvement of the upper two-thirds of the urethra, vagina, and bladder or rectal mucosa, previously categorized as stage IVA, and any lymph nodes 5 mm or smaller. Stage IIIB now includes lymph nodes larger than 5 mm. Stage IVA covers diseases fixed to the pelvic bone or regional lymph nodes that are either fixed or ulcerated, while stage IVB involves distant metastases, including those in the pelvic lymph nodes [13].

For JSGOs, the presence or absence of lymph node metastasis and the completeness of tumor excision are the most important prognostic factors [28].

Another important element is that SCCs of the vulva do not have a grade, because HPV status has much more prognostic significance than tumor grade. Indeed, there is no established grading system for vulvar carcinoma, but independent HPVs have been proven to have worse prognosis [33].

### 3.3. Early-Stage Vulvar Cancer Treatment

En bloc vulvectomy with wide margins, combined with complete inguinofemoral lymphadenectomy (IFLD), was historically used to treat vulvar SCC. Although this approach was effective in improving survival rates, it was also associated with significant short- and long-term morbidity, including wound complications, lymphedema, reduced sexual function, and adverse effects on body image.

Early-stage tumors correspond to stage I and selected II according to NCCN2024 following FIGO2021 staging. For these authors, the standard primary treatment for early-stage vulvar cancer is conservative, consisting of an individualized tumor excision with sentinel lymph node biopsy (SLNB) or IFLN evaluation. Clinicians should aim for primary tumor resection with 1−2 cm macroscopic margins. Stage IA tumors with ≤1 mm DOI need simple partial vulvectomy with pathologic study of IFLN if positive LVSI, and, in case of positive margins, re-excision if feasible with conservative surgery. Otherwise, adjuvant external beam radiotherapy is the main option. Treatment for stage IB or select stage II tumors depends on location: lateralized lesions ≥2 cm from the midline need radical partial vulvectomy and ipsilateral IFLN evaluation, while central tumors require radical partial vulvectomy accompanied by bilateral IFLN [5].

The vulvar tumors should be removed through radical local excision with histologically clear margins [24]. Although an 8 mm margin has been the target for years, evidence supporting this is limited, and the optimal margin to reduce local recurrences remains debated [37]. According to ESGO, tumors with a DOI ≤1 mm do not need groin treatment, except for positive LVSI, where lymph nodal staging should be performed. For multifocal invasive disease, each lesion may require separate radical excision. A vulvectomy might be needed for multifocal invasion with extensive vulvar dermatosis. The ideal level of radicality is still uncertain, but it is often preferred to limit radicality to preserve essential structures and functions. If the invasive disease reaches the excision margins, a re-excision is recommended.

According to ASCO, if the locoregional disease is amenable to conservative surgery, partial radical vulvectomy is indicated, with or without LN surgical staging (SLNB if T1N0 4 cm, or IFLN unilateral/bilateral according to lateralization), based on whether DOI is ≤1 mm or >1 mm. If conservative surgery is not feasible despite localized disease, primary chemo-radiotherapy (CHT-RT) is chosen. If the disease persists after CHT-RT in locoregional disease, a biopsy and potential surgery are recommended [23].

BGCS recommends primary surgical treatment to achieve negative margins while preserving pelvic function. Excision margins should extend to include adjacent differentiated VIN and/or lichen sclerosus to reduce local recurrence risk. The discrete multifocal disease may be treated with multiple local excisions, while a vulvectomy may be needed for multifocal invasion with vulvar dermatosis. If cancer extends to the margins of the excision, re-excision is preferred [25].

The IGCS consensus report for resource-limited settings recommends T1b or T2 vulvar carcinomas bigger than 2 cm from the midline with a positive inguinal lymph node to perform contralateral IFLD. For early-stage (I or II), node-negative vulvar cancer, if the tumor is less than 4 cm and margins of excised tumor are negative, after lymphadenectomy, no adjuvant treatment is required. Their recommendations are divided by consensus and by majority [26]. For further details, refer to Table 2.

For the FIGO 2021 update, surgical margins should be 2 cm to ensure pathological margins of at least 8 mm, accounting for tissue shrinkage. The deep margin should reach the inferior fascia of the urogenital diaphragm, and removal of the distal 1 cm of the urethra may be necessary. Primary closure is usually feasible, but reconstructive surgery should be considered for more significant defects [13].

According to the French 2019 guidelines, stage IA vulvar cancer (FIGO 2009) is treated with superficial partial vulvectomy, aiming for a depth of no more than 5 mm and 8 mm healthy margins after fixation (equivalent to 2 cm in fresh tissue). If the lesion is near the urethra, a 1 cm margin is acceptable if continence is preserved. Multifocal lesions require total superficial vulvectomy unless preoperative biopsies show vascular emboli, in which case a deeper excision is needed. For stages IB and II, treatment involves vulvectomy combined with inguinal lymph node surgery, with the risk of lymph node metastases increasing with lesion depth. The guidelines emphasize achieving 8 mm margins and prefer partial vulvectomy over total vulvectomy. If margins are affected or are less than 8 mm, repeat surgery is recommended, with radiotherapy considered if margins are less than 5 mm, and further surgery is not possible [27].

The JSGO treatment plan classifies vulvar tumors based on size and stromal invasion. For tumors ≤ 2 cm:—if stromal invasion ≤ 1 mm, wide local excision without lymph node dissection is recommended; if stromal invasion > 1 mm and there are lateral lesions without suspected lymph node metastasis, radical local excision with ipsilateral IFLD is indicated; for central lesions, radical vulvectomy with bilateral IFLD. For tumors > 2 cm, if resectable and not locally advanced, lateral lesions without suspected lymph node metastasis should undergo radical local excision with bilateral IFLD; if lymph node metastasis is suspected or central lesions, radical vulvectomy with bilateral IFLD [28].

For RCOG 2014 guidelines, wide radical local excision with at least 15 mm of disease-free margin is generally sufficient for primary tumor treatment. Groin node surgery should use separate incisions (triple incision technique) to reduce morbidity, as early-stage disease has a low risk of skin bridge recurrence. For unifocal tumors ≤4 cm with no clinical lymph node involvement, SLN removal is usually adequate. Tumors ≤2 cm with stromal invasion ≤1 mm (FIGO stage IA) can be managed with wide local excision alone. For tumors with DOI >1 mm (FIGO stage IB or higher) or >2 cm in diameter, groin node dissection (unilateral or bilateral) is recommended using the triple incision technique. For lateral tumors, ipsilateral groin node surgery is first, with contralateral lymphadenectomy required if ipsilateral nodes are metastatic [29].

### 3.4. Groin Nodes Assessment and SLN Biopsy

Appropriate management of groin lymph nodes is crucial for reducing mortality in early-stage VC, as groin recurrences are linked to poorer survival outcomes, even when multimodal therapies are used as “rescue” treatments [38].

SLN dissection for early VC has proven safe, accurate, and cost-effective [37], allowing greater cost-effectiveness and fewer postoperative complications, with a 10-year survival rate of 91% for patients with a negative sentinel node [27]. Included guidelines agree in citing the results of the Groningen International Study on Sentinel nodes in Vulvar Cancer V (GROINSS-V) study, which proposed the use of blue dye and Tc99m nano colloid tracers as a method for its identification [39]. Regarding indocyanine green (ICG), increasing evidence supports its use as an alternative to blue dye in SLN dissection. Even though currently, results are still mixed because they appear to be strictly related to a learning curve, and protocols are still too heterogeneous [40]. However, ESGO guidelines already recommend as feasible for SLN dissection a combination detection technique combining an isotope and either blue dye or ICG.

Lymph-nodal groin assessment implies that both inguinal and femoral nodes should be removed because dissection of only superficial inguinal nodes was associated with a higher risk of groin recurrence [41].

For NCCN 2024, IFLN evaluation is crucial and can be performed using SLN biopsy within 4 cm of lesion, or ipsilateral IFLD. If SLNs are not detected, ipsilateral IFLD should be conducted for thorough nodal assessment. Radical partial vulvectomy is recommended for anterior or posterior central vulvar lesions with bilateral IFLN surgical evaluation, which may involve SLN biopsy or bilateral IFLD [5].

ASCO 2024 recommendations indicate SLN biopsy in T1N0 stages with unifocal lesions < 4 cm or uni/bilateral IFLD depending on the anatomical location of the tumor [23].

According to updated ESGO guidelines 2023, groin treatment is recommended for tumors >T1a, based on the DOI measurement from the 8th version of the TNM classification. For non-lateralized tumors <1 cm from the midline, bilateral node evaluation is necessary. In cases of non-suspicious lymph nodes, SLN procedure is advised for unifocal tumors < 4 cm. Pre-surgery lymphoscintigraphy helps in locating SLNs, while intra-operative frozen sections are optional. If metastasis is found, bilateral IFLD is required. In case of failed drainage or inability to locate the SLN, radical lymphadenectomy of that groin is indicated. In cases of tumors ≥4 cm or multifocal disease, mandatory IFLD is required, even when lymph nodes are not suspicious. For tumors < 1 cm from the midline, bilateral IFLN evaluation is essential; when SLN criteria are fulfilled, a bilateral SNB is recommended. Then, if macrometastatic disease is found, complete IFLD is required; micrometastases or isolated tumor cells may be treated with radiotherapy, with eventual concomitant chemotherapy, omitting radical lymphadenectomy. If unilateral metastasis is found, further treatment is usually limited to the affected groin. In cases of unilateral failure of drainage or undetected SLN, IFLD should be performed [24].

For 2023 BGCS guidelines, groin treatment is required if the primary tumor’s depth exceeds 1 mm; SLN biopsy is preferred for small, unifocal tumors <4 cm with no clinical or radiological signs of lymph node metastasis, >1 mm depth, with no adjacent organ involvement, provided accurate injection and analysis are possible, and the tumor does not involve adjacent structures. IFLD is recommended for tumors ≥4 cm or multifocal disease, with deep femoral nodes removed and the saphenous vein preserved when possible [25].

According to FIGO 2021, separate incisions are indicated for resection of the primary tumor, and groin lymph nodes, with an improved healing. Stage IB or resectable Stage II cancers require IFLD. Bilateral groin node dissection is necessary for tumors near or crossing the midline, large lateral tumors (>4 cm), or positive ipsilateral nodes. SLN procedure is indicated for the same BGCS conditions [13].

IGCS 2022 does not contain any specific indications regarding the SLN. It presented considerable heterogeneity of responses about this topic and needed to reach a target to provide a management direction [26].

According to French guidelines, lymph node staging is recommended for tumors classified as ≥ IB; the SLN technique is standard for unifocal tumors <4 cm with no lymph node involvement, performed unilaterally for lateral lesions and bilaterally for medial lesions, and its detection should use colorimetric, isotopic methods, or ICG fluorescence; IFLD should target both superficial and deep medial nodes, preserving the saphenous vein and fascia. If lymph node involvement is detected, contralateral IFLD is required [27].

SLN biopsy in Japan, given the declared low level of experience, should still be regarded as an experimental method. It can be used to prevent the need for an IFLD when there is no clinical suspicion of metastasis to the inguinal lymph nodes. Since these guidelines were published in 2015, they certainly deserve updating. IFLD can be skipped for tumors measuring ≤2 cm and with stromal invasion ≤1 mm. This procedure involves the removal of both superficial and deep inguinal lymph nodes. Ipsilateral lymphadenectomy may be sufficient for laterally located tumors that are ≤2 cm in size [28].

According to RCOG, SLN is suitable for primary squamous VCs <4 cm, unifocal, with no lymph node metastasis signs, and no safety issues with Patent Blue dye or technetium-99. Informed consent is required, with follow-up appointments every two months for the first year. If SLN is not located, a complete IFLD should be considered, and patients should be informed during consent. Superficial groin node dissection alone is associated with higher groin node recurrence risk; preserving the long saphenous vein can reduce complications. However, if a complete lymphadenectomy is needed, both superficial inguinal and deep femoral nodes should be removed [29]. In stage IA, it could be avoided.

Clear indications for treatment in case of stage IA with positive LVSI are diffusely lacking.

Table 2 summarizes all these findings.

### 3.5. Advanced-Stage Vulvar Cancer Treatment

Locally advanced vulvar cancer is defined differently across guidelines. NCCN defines it as tumors unresectable without removing adjacent structures. Treatment involves external beam radiation therapy (EBRT) with concurrent chemotherapy. If lymphadenectomy is performed, it helps plan radiation, covering the primary tumor, groin, and pelvic nodes if positive nodes are found [4]. The GROINSS-V II trial examined indeed the effectiveness of groin radiation in place of inguinal lymphadenectomy for patients with a single positive SLN [42]. Interim results showed increased recurrence rates in patients with SLN metastasis larger than 2 mm or with extranodal extension. Following a protocol amendment, patients with SLN metastasis ≤2 mm received radiation, while those with more extensive metastases underwent lymphadenectomy. The study concluded that radiation is a safe alternative for more minor vulvar cancer cases with SLN metastasis ≤2 mm. However, for more extensive metastases, radiation alone resulted in more groin recurrences than surgery [42].

For ASCOs, VCsare tumors invading nearby perineal structures or presenting with fixed or ulcerated lymph nodes. ASCO recommends upfront chemoradiotherapy for inoperable cases, with possible subsequent surgery for persistent disease [23].

In ESGO recommendations, advanced stage is defined as clinical >T3 and/or >N2 and primary chemoradiotherapy is recommended for unresectable cases, and in stage IVB with pelvic lymph node involvement, targeting the primary tumor site as well as bilateral inguinofemoral, and pelvic nodes [24,25,26,27,28,29,30,31,32,33,34,35,36,37,38,39,40,41,42,43,44,45]. Post treatment, in cases of clinical complete response, a biopsy of the tumor bed may be considered to confirm pathological response (pCR). Patients with no clinical residual tumor should be closely monitored, while those with residual disease may require surgery or further treatment depending on the tumor status. If residual disease is unresectable, additional radiation therapy, systemic therapy or best supportive care should be considered. Moreover, neoadjuvant chemoradiotherapy may be considered, but its role is debated [42,43,44]. Additionally, after a multidisciplinary evaluation, neoadjuvant platinum-based chemotherapy might be considered for selected patients who are not suitable or fit for upfront surgery or primary chemoradiotherapy. After 3–4 cycles of chemotherapy, re-staging and reassessment for definitive treatment should be conducted [24]. It should also be considered if exenterative surgery should be the option otherwise.

The BGCS recommends primary CHT-RT therapy for patients with tumors too extensive for surgery, those needing exenterative surgery with permanent stoma formation, or those unfit for anesthesia. For locally unresectable tumors, definitive CHT-RT is preferred [25].

IGCS 2022 recommendations by consensus indicated conventional 2D radiotherapy, including the cobalt technique if necessary, for locally advanced vulvar cancer, namely, pelvic node disease. Concomitant CHT-RT is the favored treatment for patients with unresectable vulvar cancer [26]. Instead, guidelines by the majority recommended radical vulvectomy with bilateral IFLD followed by radiation as the best treatment if chemotherapy is not available. Best supportive care should be offered for patients with poor geriatric scores and/or poor performance status when radiation therapy or chemotherapy is not available.

FIGO 2021 defines management as individualized and involves a multidisciplinary team. Pretreatment assessment of groin nodes is crucial. When surgery is not viable, primary chemoradiation targeting the primary tumor, groin, and pelvic nodes is recommended. Enlarged groin and pelvic nodes should be removed if possible, followed by postoperative radiation. Biopsy and primary radiotherapy should be used for ulcerated or fixed nodes, with potential surgery for incomplete responses. Optimal treatment for advanced VC includes excising the primary tumor with clear margins, and if necessary, radiotherapy or chemotherapy may be used, especially if exenteration is required [13,43].

French guidelines emphasize no established criteria for determining non-resectability; instead, this determination is primarily based on the expertise and experience of the surgical team. They advocate primary radiation for inoperable cases or when exenteration is needed. For resectable tumors, radical total vulvectomy with bilateral IFLD followed by radiation may be used. Neoadjuvant chemoradiotherapy can be employed based on post-treatment evaluation [26]. In complete clinical regression, directed biopsies should be performed on any suspicious areas to confirm regression. If the biopsies are negative and surgery is not performed, additional external radiotherapy may be considered. If microscopic residue is found, an excision will be carried out based on the preoperative samples. For partial regression allowing surgical excision, the tumor area must be excised widely and radically in depth. Additional radiotherapy may be discussed if the excision margins are less than five millimeters.

Japanese guidelines surprisingly include surgery, recommending pelvic exenteration or radical vulvectomy for resectable cases, possibly combined with chemoradiotherapy [28]. For tumors larger than 2 cm in diameter with a locally advanced but resectable lesion, the surgical options include [28]: radical vulvectomy with distal urethral resection, inferior colpectomy, bilateral IFLD, exenteration, that may be combined with chemotherapy, and/or preoperative radiotherapy [28].

RCOG emphasizes meticulous preoperative planning for advanced disease, with radical excision to be coordinated with a plastic surgeon in a EUA or, alternatively, radiotherapy either with curative intent or neoadiuvant to reduce tumor volume if necessary. Groin node dissection is advised for suspicious nodes, and reconstructive techniques may be used to minimize scarring and morbidity. Wide radical local excision with a minimum of a 15 mm disease-free margin may be employed, though some tumors will necessitate a radical vulvectomy. Suppose these surgical approaches pose a risk of sphincter damage, potentially leading to urinary or fecal incontinence. In that case, radiotherapy should be considered either with curative intent or to reduce tumor volume, allowing for less destructive surgery [29]. Reconstructive surgical techniques should be utilized to facilitate primary surgical closure and to minimize morbidity associated with scarring.

The summary of these results is shown in Table 3.

### 3.6. Adjuvant Therapy

The current standard of care for patients with SNL metastases involves additional treatment with IFLD. Adjuvant radiotherapy following IFL is recommended for patients with metastatic lymph nodes and/or extracapsular extension.

NCCN guidelines highlight the need for further research on adjuvant treatments, given the rarity of the disease, and recommend therapy based on tumor presence at the primary site and groin, with considerations for risk factors like tumor size, margins, and invasive patterns [44]. Patients with positive margins should undergo re-excision, or if resection is not possible without affecting the proximal urethra, bladder, or anus, adjuvant radiotherapy (RT) should be considered.

The ESGO panel advises that patients with negative margins after primary surgery either undergo monitoring or receive radiation therapy to the vulvar site, depending on local risk factors such as close but clear pathological margins with extensive LVSI or perineural involvement, as well as lymph node involvement. For patients with persistent positive margins following re-excision, RT is recommended. In cases of stage IB and II vulvar cancer, adjuvant therapy is determined by primary tumor characteristics.

After SNLB, if micrometastases (<2 mm) or isolated tumor cells are present, radical surgery may be avoided in favor of adjuvant RT, which has lower morbidity. Following lymphadenectomy, the discovery of more than one metastatic lymph node or extracapsular spread warrants adjuvant RT, potentially combined with concurrent chemotherapy.

ASCO emphasizes that adjuvant RT to the vulva can result in significant side effects, including skin, genitourinary, gastrointestinal, and perineal complications. Due to the lack of randomized controlled trials confirming the benefit of adjuvant RT after surgery, it is generally reserved for cases with positive margins where re-excision is not an option [23]. Consequently, the presence of positive margins when organ-conserving re-excision is unfeasible is the only widely accepted indication for adjuvant RT [46].

Key risk factors from the National Cancer Database, such as lymph node involvement, large tumor size (>4 cm), multifocality, and stromal invasion > 5 mm, help guide RT decisions [47]. While a dose of 54–56 Gy may benefit involved margins, exceeding 60 Gy is discouraged due to potential complications and no evidence that adding chemotherapy improves outcomes without lymph node involvement. Additionally, no evidence adding concurrent chemotherapy to adjuvant RT improves outcomes for patients without IFLN involvement [23]. The ESGO guidelines outline three scenarios for adjuvant radiotherapy in vulvar cancer treatment as follows:

(1) Postoperative radiotherapy to the vulva is recommended when the invasive tumor reaches the excision margins and further surgery is not possible. Radiotherapy may be considered individually for cases with clear but close margins and other risk factors (e.g., lymph node involvement, LVSI, perineural invasion).

(2) For SLN metastases smaller than 2 mm or isolated tumor cells (ITC), RT can replace IFLD, offering fewer long-term side effects.

(3) RT is recommended for patients with more than one metastatic lymph node or extracapsular spread, possibly with radiosensitizing CHT. Treatment should be individualized and completed within 104 days post surgery, using intensity-modulated radiation therapy (IMRT) to avoid delays or breaks [24].

The BGCS guidelines recommend starting adjuvant (chemo)radiotherapy within 6 to 8 weeks after surgery [19]. Postoperative radiotherapy is advised in the following situations:

– When the primary tumor has positive excision margins, further surgery is impossible.
– For pathological margins under 2 mm, even though there is no consensus on the optimal threshold, decisions should be individualized and discussed in a multidisciplinary team, considering factors like patient health, location of margins, and the need for groin or pelvic radiotherapy.
– After IFLD, if more than one metastatic lymph node or extracapsular involvement is present.
– After SLN biopsy, if micrometastasis is detected.

The IGCS 2022 guidelines for vulvar cancer treatment in resource-limited settings suggest the following: contralateral IFLD is recommended for T1b or T2 tumors more than 2 cm from the midline with a positive homolateral inguinal lymph node. For tumors less than 4 cm and within 2 cm from the midline, contralateral IFLD should be considered if unilateral uptake is seen on lymphoscintigraphy. No adjuvant treatment is needed for early-stage, node-negative vulvar cancer with tumors less than 4 cm and negative margins. RT can be used if only cobalt machines are available. Adjuvant chemoradiation should be offered for patients with macroscopic nodal disease or multiple micrometastatic nodes after lymphadenectomy [26].

According to FIGO2021, critical prognostic factors for vulvar cancer include the number and size of groin node metastases and the presence of extracapsular spread [14,48]. Patients with a single, small lymph node metastasis generally have a favorable prognosis with IFLD alone if there is no extracapsular spread and usually do not benefit from additional RT.

For patients with positive groin nodes requiring RT, the criteria for pelvic and groin irradiation include extracapsular spread or two or more positive groin nodes. For advanced vulvar cancer, RT should cover the pelvis, inguinal nodes, and vulva, with a minimum dose of 50 Gy to the inguinal nodes. It should include the inguinal, external, and internal iliac lymph nodes and extend the field if there are numerous or significant positive nodes or suspected pelvic node metastases [49].

The French guidelines suggest adjuvant treatment for stage IB and II vulvar cancer, recommending RT for cases involving the first two lymph nodes and/or extracapsular spread. The RT should cover the pelvis, including external and internal iliac nodes and the groin, with a dose of 45 to 50 Gy. Concurrent chemotherapy may be added based on the risk of recurrence and patient comorbidities [27].

The JSGO guidelines recommend postoperative RT for VC cases with metastatic inguinal lymph nodes after radical vulvectomy and IFLD. Enlarged lymph nodes should be resected and examined histologically for metastases if possible. Adjuvant RT of the primary site is advised if the excision margin is less than 8 mm or if there is advanced vascular space invasion. For the groin and pelvis, RT is recommended when there are two or more metastases or extracapsular invasions. However, postoperative RT may be omitted if there is only one inguinal lymph node metastasis without extracapsular spread [28].

The RCOG guidelines outline RT should be considered if there are two or more lymph nodes with microscopic metastases in either groin or if there is a complete replacement or extracapsular spread in any lymph node. As of their 2014 publication, there was no conclusive evidence on whether RT should be administered to either side. Nonetheless, they recommended that treatment cover both the groin and the pelvic nodes [29].

### 3.7. Follow-Up

The follow-up protocols for VC vary among international guidelines but generally aim to monitor recurrence and manage long-term effects. The general approach foresees follow-up should address both physical and psychological impacts of treatment, including lymphoedema management and psychosexual counseling. Most recurrences occur within the first 1–2 years, though some may appear after 5 years [50]. Overall, follow-up schedules are tailored based on individual risk and treatment response, emphasizing early detection of recurrence and managing treatment-related side effects.

The IGCS report 2022 on guidelines for managing VC in resource-poor areas needs to include recommendations for the follow-up of treated patients [26].

NCCN guidelines recommend follow-up every 3–6 months for the first 2 years, then every 6–12 months for 3–5 years, and annually after that. High-risk patients may need more frequent assessments, such as every 3 months during the first 2 years, compared to those with low-risk disease, who may be assessed every 6 months. Imaging and lab tests are used based on symptoms or suspicious findings. However, the role of ultrasound for post-treatment follow-up in stage IB or higher is emphasized. Patient education regarding suggestive recurrence symptoms should be performed [5].

ASCO guidelines suggest clinical exams every 3–4 months for the first 2 years, then every 6 months. Imaging (e.g., MRI, PET/CT) should be performed 3–4 months post treatment to confirm remission. They emphasized the need for BSC, consisting of pain management, wound care, management of sexual dysfunction, psychosocial support, and palliative care [23].

ESGO guidelines move from the consideration of an optimal follow-up schedule for VC, which remains uncertain and should be personalized in terms of intensity, duration, and procedures based on individual risk assessments [24]. Therefore, it advises follow-up 6–8 weeks after treatment, every 3–4 months for 2 years, biannual or annual visits from years 3–5, and long-term surveillance for ongoing issues or side effects.

Agreeing on the lack of standardization, the BGCSs state that recurrence rates or new foci are common, particularly in patients with a background of differentiated vulvar intraepithelial neoplasia (dVIN) or lichen sclerosus. Patients with HPV-related unifocal disease are at lower risk of recurrence and in the absence of new areas of uVIN developing during follow-up, discharge to primary care, with emphasis on the need for prompt referral if a new lesion develops, may be considered after five years [25]. However, those with recurrent disease, HPV-independent VSCC, or multifocal disease may require lifelong follow-up. BGCS recommends follow-up every 3–6 months for the first 2 years, then 6–12 months up to 5 years, with more frequent visits for high-risk or recurrent cases. Imaging at 10–12 weeks post treatment is suggested.

FIGO 2021 recommends a follow-up schedule for all gynecological malignancies, including VC. Patients should be monitored every 3 to 6 months for the first 2 years, then every 6 to 12 months until 5 years post treatment. Follow-up visits should include symptom review, examination for recurrence or treatment-related effects, and clinical assessments. Unique to their guidelines, FIGO notes that elevated pretreatment serum concentrations of squamous cell carcinoma antigen (SCC-Ag) may be an independent prognostic factor and can be used as a tumor marker for some VC patients. However, specific criteria for its use need to be more detailed [13].

French guidelines for follow-up include complete perineal and pelvic examinations and palpation of inguinal lymph nodes every 3 to 4 months for the first year, then every 6 months for the next 2 years, and then annually. For locally advanced or lymph node-involved cases, a thoracic–abdominopelvic scan is recommended every 6 to 12 months for 2 to 3 years. PET scans should be used if recurrence or metastasis is suspected. For advanced stages treated with RT or CHT-RT, imaging is recommended 6 to 8 weeks after treatment if surgery is considered. Otherwise, imaging or a PET scan is advised 4 months post treatment [27].

JSGO guidelines for follow-up after VC treatment suggest scheduled visits every 1 to 3 months for the first 2 years, every 6 months from the third to the fifth years, and then annually. During follow-up, it is essential to conduct medical interviews, physical inspections, palpations, and perform tests such as cytology, biopsies, chest X-rays, tumor marker assessments, and CT scans. Monitoring should address not only recurrence but also potential complications [28].

RCOG 2014 follow-up considers that since salvage therapy typically relies on further excision or radiotherapy, it is logical to aim for early recognition of recurrence. For this reason, most centers follow a regimen of every 3 months for the first year, every 6 months for the second year, and annually after that. Late recurrence is uncommon but can occur. Thus, follow-up may be necessary for many years [29].

A summary diagram can be found in Table 4.

### 3.8. Recurrence of Disease Treatment

Recurrent VC can present as local vulvar, inguinofemoral, or distant recurrences, individually or together. About 12–37% of women with VC experience recurrence, typically within the first two years after initial treatment. Those with p53-mutated tumors (not linked to HPV) and patients with involved lymph nodes face the highest risk of recurrence [51].

A multicenter case series of 500 patients with vulvar cancer found that most recurrences were local, with 53.4% being vulvar, 18.7% inguinal, 14.2% multi-site, 7.9% distant, and 5.7% pelvic. Five-year survival rates varied significantly by recurrence location: 60% for vulvar recurrences, 27% for inguinal or pelvic recurrences, 15% for distant recurrences, and 14% for multi-site recurrences [52].

For suspected vulvar cancer recurrence, the NCCN panel advises imaging with CT or FDG-PET/CT and possibly a biopsy. Treatment varies by recurrence site and past treatments. For vulvar-only recurrences without prior radiation, options include radical vulvectomy with possible IFLD or pelvic exenteration for central recurrences.

Additional treatment is guided by margin and nodal status. For negative margins and nodes, observation or RT is appropriate. In cases with positive margins but no nodal involvement, options include re-excision or RT with or without brachytherapy and/or concurrent chemotherapy. For patients with negative surgical margins but positive IFLNs, RT with or without concurrent chemotherapy is recommended. In cases where both margins and IFLNs are positive, the panel recommends a combination of RT, brachytherapy, concurrent chemotherapy, and/or re-excision as appropriate. For non-surgical treatment of recurrence, RT with or without brachytherapy and/or concurrent chemotherapy is recommended. In cases with a significant residual tumor, resection may be considered. For nodal or distant recurrences, treatment depends on the extent and location of the disease. Systemic therapy, selective RT, or palliative care may be necessary, especially if multiple pelvic nodes or distant metastases are involved. Systemic options include carboplatin, paclitaxel, and bevacizumab, with PD-1 inhibitors as a second-line option for specific cases. In cases of isolated IF/pelvic recurrence that were previously irradiated, resection followed by systemic therapy can be an option. If no prior RT was administered, RT with or without concurrent chemotherapy is recommended [5].

Instead of providing explicit treatment recommendations for relapses, the ASCO guidelines conduct a literature review of the latest scientific evidence. However, for metastatic disease outside the pelvis, they recommend systemic platinum-based therapy in combination with PD-1 inhibitors (for patients with a combined positive score (CPS) >1) with or without bevacizumab [23].

ESGO recommends a comprehensive approach involving a multidisciplinary team at a specialized center. Initial steps include a thorough vulvar examination, biopsies of suspicious areas, and imaging (ultrasound, MRI, CT, or 18F-FDG-PET) of the chest, abdomen, and pelvis [24]. Biopsies are also advised for suspected nodal or distant recurrences, with early palliative care referral for incurable cases. Treatment varies by recurrence type: radical excision of the affected area is recommended for local recurrence. If margins are involved, re-excision or postoperative RT (if not previously performed) may be needed. As recurrence could be classified as a second primary disease, surgical groin re-staging should be considered in case of clinically negative IFLN. Locally advanced recurrent cases, never treated by RT, may require definitive (chemo)RT. Alternatively, pelvic exenteration can be evaluated according to patient performance status. IFLD or debulking is recommended for IF nodal recurrence, followed by (chemo)RT, if no prior RT was delivered. This is also advised for pelvic nodal recurrence, with possible debulking of enlarged nodes before treatment. Options for patients with previous RT include surgical resection, stereotactic RT, and systemic therapy. This last one is also an option for distant metastases, with stereotactic RT or surgery for oligometastatic cases. PD-1 inhibitors are suggested as first-line therapy for select patients with specific biomarkers in combination with platinum-based chemotherapy [24].

According to BGCS, surgical re-excision should be considered for patients with local or IFLN relapses if the disease is amenable to surgery, mirroring the initial treatment approach. Imaging studies are recommended before treatment to tailor the treatment plan. SLN biopsy may be an option for recurrent disease if the new invasion area qualifies, though data on its safety and efficacy are limited. For patients ineligible for surgery, palliative chemotherapy, radiotherapy, or a combination should be assessed based on prior treatments, patient preferences, and overall health. Systemic therapy may be considered for distant metastases, but specific treatment protocols are not well established [25].

The IGCS recommendations suggest that salvage surgery should be considered for patients with potentially resectable local recurrences and no suspected lymph node involvement, provided they have no significant comorbidities and have previously undergone initial surgery [26]. In cases where patients have comorbidities, cisplatin and radiation therapy are contraindicated. Salvage surgery is advised for potentially resectable local recurrences regardless of prior treatment, including those who have previously undergone radiation. For clinical lymph node recurrences, salvage surgery is recommended if resectable, with treatment choices depending on prior therapy and patient condition: cisplatin-based therapy for patients without comorbidities who received only surgery initially; carboplatin and paclitaxel for those who had adjuvant radiation or chemoradiation; and radiation therapy for those with comorbidities or contraindications to cisplatin [26].

FIGO2021 and French guidelines merely considers the therapeutic alternatives that may be available (surgery, (CHT)RT, neoadjuvant or palliative chemotherapy, a targeted agent (if available), or BSC) [13,14,15,16,17,18,19,20,21,22,23,24,25,26,27].

Re-excision is a therapeutic option also for JSGOs in localized recurrence after surgery. Concurrent CHT-RT is considered for unresectable local recurrences or infiltrating adjacent organs, provided those areas have not been previously irradiated. Systemic CHT is recommended for pelvic recurrences accompanied by distant metastasis or multiple lesions. BSC is considered when no other effective treatment options remain [28].

According to the oldest RCOG guidelines, groin recurrence is associated with a significantly poorer prognosis and poses management challenges. For women who have not previously received groin irradiation, RT (with or without surgery) is the preferred treatment option. However, for those who have already undergone irradiation, treatment options become considerably limited, and palliative care, which may include surgery, should be considered. In cases where women have had both surgery and RT to the groin, the palliative care team should be involved promptly after groin recurrence is confirmed [29].

A summary of all these above-mentioned indications is available in Table 5.

## 4. Discussion

Managing vulvar tumors is a complex challenge that requires a multidisciplinary approach involving gynecological oncologists, psycho-oncologists, and plastic surgeons, especially for postoperative reconstruction. Studies like the Vulcan Study highlight the importance of such collaboration in improving patient outcomes [52]. However, a comparative analysis of European, American, and Asian guidelines reveals substantial differences in the management of vulvar cancer, particularly in diagnostic protocols, staging, and treatment strategies.

Furthermore, there must be a differentiation between national and supranational guidelines because they adapt to different realities.

On the diagnostic side, some remarks deserve to be made about biopsy, a key diagnostic element in vulvar cancer. While all guidelines recognize its importance, differences exist in the recommended type. ESGO, BGCS, and RCOG emphasize incisional or punch biopsy over excisional biopsy, as the latter can complicate staging and treatment decisions. In contrast, other guidelines such as NCCN, ASCO, and IGCS leave the choice of biopsy type to the clinician’s discretion.

Additionally, complete excision of small lesions can enhance diagnostic accuracy and, especially if there is a long wait for surgery, may help halt disease progression and reduce the risk of lymph node metastasis. Indeed, no current guidelines specify a maximum time frame between diagnosis and the initial surgical treatment. Moreover, none of the guidelines explicitly states that the gynecologist oncologist dealing with the vulva should have the skills to perform at least simple plastic surgeries: in reality, plastic surgeries are few and far between and cannot take on the whole burden of the VC, except in a few centers.

The need for multidisciplinary collaboration in surgical planning is explicitly addressed by BGCS and NCCN, although it is less emphasized in other guidelines, despite its recognized value in ensuring optimal patient outcomes. ESGO stresses the importance of clinical documentation, including photographic images of lesions before treatment, a practice also supported by British and American guidelines, though less clearly in other sources.

There is general consensus on the need for cervical and vaginal screening for HPV-related tumors in the diagnostic setting, but IGCS guidelines, tailored for low-income settings, do not include this as standard practice due to lower cervical cancer screening rates in these regions. Imaging techniques are another area of difference between guidelines.

A surprising aspect is that ESGO stands alone in fully adopting the VITA 2021 criteria, despite their comprehensive approach to ultrasound diagnostics.

*Assessment of Inguinal-Femoral Lymph Nodes (IFLN):*

The 2023 BGCS guidelines acknowledge the role of ultrasound in evaluating inguinal-femoral lymph nodes. However, it is considered secondary to CT for lymph node assessment. Despite valuable contributions from the VITA group, most international guidelines have not yet adopted these criteria, resulting in variability in clinical practice [31].

*Diverging Approaches to Lymph Node Assessment:*

The assessment of inguinal lymph nodes is widely recognized as critical in treatment decisions, but notable differences persist. For instance, the following:

The ESUR guidelines recommend:

MRI for tumors with stromal invasion greater than 1 mm or those larger than 4 cm.

CT for locally advanced FIGO stages III—IVA or suspected distant metastases.

Ultrasound primarily for evaluating IFLN and guiding biopsies.

Despite these recommendations, the integration of imaging modalities into treatment protocols varies across different guidelines.

*Variability in Level II Radiological Imaging:*

There is significant inconsistency in Level II imaging recommendations among various guidelines, even with ESUR’s specific suggestions [32]. These include the following:

MRI for tumors with:

Stromal invasion greater than 1 mm.

Diameters up to 4 cm.

Proximity or direct involvement of adjacent anatomical structures.

CT for:

Locally advanced FIGO stages III–IVA.

Suspected distant metastases.

Ultrasound for:

Assessing inguinofemoral lymph nodes.

Guiding biopsies of suspected lesions.

*Viral Screening in Vulvar Cancer Management:*

Screening for viral infections, particularly HIV, is strongly encouraged in the management of vulvar cancer. This recommendation stems from the increased risk associated with immunodeficiency, especially in cases linked to HPV [51].

Various guidelines, including ESGO, highlight vaccination as part of primary prevention for vulvar cancer, though implementation may vary across countries [13,25].

A significant discrepancy among guidelines is the use of different staging systems, such as FIGO 2009, FIGO 2023, and the 8th edition of AJCC TNM. Misalignment between TNM and FIGO 2021 stages has led to differing approaches to early-stage vulvar cancer. For example, NCCN classifies some cases as stage II, while ESGO follows the TNM system, aligning with FIGO stage III. This affects treatment decisions, such as excision methods. While NCCN recommends simple partial vulvectomy for stage IA tumors, ESGO advises radical local excision. ESGO also emphasizes that R0 resection should include removal of VIN, which may necessitate vulvectomy for function preservation.

Variability also arises from differing criteria for measuring depth of invasion (DOI). DOI directly influences the extent of surgery, balancing the need for effective treatment with minimizing morbidity. Precisely, DOI ≤ 1 mm is considered a low risk for lymph node involvement. Therefore, surgery limited to wide local excision with clear margins, avoiding unnecessary lymph node dissection should be considered. Differently, for DOI > 1 mm, a higher risk of lymphatic spread must be taken into account necessitating SLNB or full IFLD, with a more aggressive surgical planning to ensure complete staging and reduce recurrence risk. The distinction in pathological evaluation of depth creates a divide between guidelines that use this criterion [5,24] and those that do not [27,29]. Surgical management for staging commonly includes excision (from simple excision to vulvectomy), lymph node staging (including inguinal-femoral lymphadenectomy), and sentinel lymph node biopsy (SLNB) when no signs of nodal involvement are present.

An active area of research is the comparison of endoscopic techniques to traditional open inguinal-femoral lymphadenectomy. A pilot study suggests that endoscopic methods may reduce post-surgical morbidity while maintaining oncological efficacy [53]. Though the SLN technique is widely accepted, the approach to inguinal surgery remains far from minimally invasive. Research also focuses on reconstructive strategies to address the functional impact of vulvar and inguinal surgery, which significantly affects quality of life. Reconstructive surgery is under-represented in the guidelines, with ESGO offering only general guidance and BGCS providing more detailed recommendations.

BGCS guidelines summarize various skin and tissue reconstruction options, such as split skin grafts, which are simple but prone to contracture, and dermal replacements, which are more flexible but still in development. Local flaps, such as rhomboid and lotus petal flaps, are suitable for small defects but may be thicker than needed. Larger defects may require distant flaps like gracilis, rectus abdominis, and anterolateral thigh flaps, which carry risks like devascularization and complications at donor sites. The limited focus on reconstructive strategies in guidelines likely reflects the fact that pelvic exenteration is generally reserved for second- or third-line treatment due to its functional consequences. Specific guidance on reconstructive strategies exists in the literature, particularly for recurrent vulvar cancer in preirradiated patients. Arcieri et al. proposed a management algorithm detailing the appropriate flap types for reconstructive surgery following second- and third-degree exenterations, particularly in cases involving preirradiated patients, which is common in recurrent vulvar cancer contexts [54].

Another key observation from the comparison of guidelines is the frequent emphasis on “resectability” and “non-resectability,” which guide decisions between eradication surgery and alternative therapies such as radiation or chemoradiation. None of the recommendations offer objective and definitive criteria that could assist gynecological oncologists and multidisciplinary teams to make initial treatment decisions [55]. This is undoubtedly a valuable concept for thought that should stimulate future research.

However, no current guidelines set a minimum experience requirement for the surgical team or practitioner, such as a benchmark for the number of cases per year. This is crucial not only for the technical aspects of procedures (e.g., SLNB) but also for the expertise needed to accurately assess whether a lesion is operable.

The perceived limited role of exenterative surgery might contribute to the absence of well-defined recommendations for the postoperative management of these patients. Such cases often require advanced and specialized care, even if this topic is frequently underestimated among guidelines. Indeed, only BGCS discusses using vacuum-assisted closure (VAC) dressings. This tool could be handy in surgical wound management, but its effectiveness is limited by difficulties securing a proper seal due to local anatomical features [25]. VAC dressings represent a challenging instrument for inguinal wounds, as they may be easily infected and a long-lasting opening for the anatomical site. A Cochrane review has suggested that negative pressure could reduce the healing time for secondary intention wounds, although evidence in support is still limited [56]. Innovative therapeutic arms are emerging to drive prolonged healing processes and offer new avenues for investigation [21].

In the management of advanced and metastatic vulvar cancer, immunotherapy is gaining importance. The KEYNOTE-158 study explored the efficacy and safety of pembrolizumab, one of the most promising PD-1 inhibitors, in patients with advanced vulvar squamous cell carcinoma (VSCC) who had previously been treated with standard therapies. The study aimed to assess whether immunotherapy could offer a viable treatment option for patients with this challenging, often refractory disease. It showed an overall response rate (ORR) of 16%, indicating that a modest number of patients experienced partial or complete responses, especially those with HPV-positive tumors; median progression-free survival (PFS) was 2.1 months, with some patients showing durable responses lasting over 12 months. Even if the results showed that pembrolizumab provides only a modest benefit overall, it highlights an important shift toward immunotherapy as a potential treatment option for advanced vulvar cancer, especially for HPV-positive cases, by opening up the possibility of using immune checkpoint inhibitors in second-line or subsequent therapies, offering a new hope for patients with limited treatment options [57].

As proof of the aforementioned, pembrolizumab has already been incorporated into the 2024 NCCN guidelines as a second-line treatment for PD-L1-positive relapses [5].

Electrochemotherapy (ECT) is also gaining attention for its efficacy in palliative care, especially in patients who have undergone multiple treatments. ECT enhances the effect of cytotoxic drugs like cisplatin and bleomycin through electrical pulses and has shown promise in improving quality of life, although it is primarily used in palliative care [12,58]. Studies have demonstrated that ECT is effective in achieving local tumor control in vulvar cancer patients. For instance, a study reported an overall response rate of approximately 70% in patients treated with bleomycin-based ECT in a palliative setting [12].

Another study highlighted that ECT is a feasible and safe technique for treating vulvar cancer recurrences, with outcomes comparable to surgical interventions [59]. Research is even exploring the potential of combining ECT with other treatment modalities, such as immunotherapy, to enhance therapeutic outcomes [12]. However, currently, ECT is only mentioned in ESGO guidelines for a palliative care setting when other treatments are no longer viable.

Agreement on the lack of standardization in follow-up protocols was obtained: each scientific society felt free to offer its schedule. Notably, only FIGO mentioned the potential role of SCC-Ag in the early detection of relapse [13].

There are no recommendations on the management of lichen when causing HPV-independent VC at the end of cancer treatment. Unclear recommendations are also for the post-surgical management of inguinal lymphadenectomy. What is even more surprising is that there are no defined reliability criteria for inguinofemoral lymphadenectomy as well as no recommendations for anatomical markers.

A reference to the importance of the geriatric assessment in the overall counseling of the patient is emphasized locally in the English, French, American ASCO, and international European ESGO guidelines, although not all of them clearly indicate it in the pretreatment phase.

In summary, while there is broad agreement on many aspects of vulvar cancer management, significant variability exists in the diagnosis, staging, treatment, and postoperative care approaches across different guidelines. This inconsistency underscores the need for further research and standardization to improve patient outcomes.

Beyond all these considerations, in the future perspective, prominent focus areas that will grow more are immunotherapy and the insights that research into the tumor microenvironment in preclinical models will provide [55]. Today, there is indeed emerging evidence suggesting that myeloid and/or lymphoid infiltrates could predict excellent therapeutic responses [15].

## 5. Conclusions

This comparative analysis of international guidelines for vulvar cancer management highlights significant variability in recommendations, particularly regarding diagnostic techniques, surgical treatment options, and follow-up protocols.

The need for a highly specialized, multidisciplinary approach is widely recognized, but its implementation is inconsistent across different scientific societies. For example, only certain guidelines clearly emphasize collaboration between gynecological oncologists and plastic surgeons to optimize surgical treatment and postoperative reconstruction, while others do not directly address this necessity. Moreover, recommendations on the use of ultrasound for lymph node evaluation, in line with the VITA group consensus, are incorporated only in the most recent guidelines, such as those from ESGO, but are still absent in other key guidelines. This raises questions about how new evidence is adopted and integrated into clinical protocols.

Treatment options for advanced stages of vulvar cancer, including immunotherapy and electrochemotherapy for palliation, remain under-represented despite growing evidence of their effectiveness. The lack of robust data limits the uniform inclusion of these options in international protocols, underscoring an opportunity for future clinical trials and a more harmonized set of guidelines.

Finally, the management of recurrences and follow-up protocols varies greatly and lacks standardization, suggesting an opportunity to develop risk-based guidelines that consider both prognostic factors and the psychological needs of patients.

In conclusion, this review underscores the urgency of regular updates and greater alignment among international guidelines to ensure that vulvar cancer care is both evidence-based and responsive to the complex needs of patients.

## Figures and Tables

**Table 1:** Diagnostic work-up.

	2024 NCCN	2024 ASCO	2023 ESGO	2023 BGCS	2022 IGCS	2021 FIGO	2019 French	2014 RCOG
**Clinical exam**	GV.	GV.	GV; in advanced stage: LN and rectal assessment.	GV.	NM.	GV.	GV + LNI assessment. (*)	GV + pubic and ischial bones assessment.
**Clinical photos**	NM.	NM.	Yes.	Yes, vulvoscopy before and after biopsy.	NM.	Yes, on pathological piece removed.	NM.	Yes.
**Biopsy**	ND.	ND.	Incisional/punch.	Incisional/punch.	NM.	Incisional/punch.	ND.	Incisional/punch. Excisional if symptoms and definite suspicion.
**Cervico-vaginal citology**	Yes, cervix and vagina.	Yes.	Yes, cervix and vagina.	Yes.	NM.	Yes and colposcopy.	Yes, with colposcopy.	No.
**EUA**	If indicated.	NM.	NM.	For large lesions, to be performed by gyneco-oncologist and plastic surgeon for surgical planning.	NM.	NM.	NM.	NM.
**US**	NM.	NM.	For IF nodes.	If SLN is considered, for groin. *		NM.	To evaluate groin nodes. *	NM.
**CT**	Yes, to evaluate disease extension. *	Abdomen chest. *	Systemic staging (including distant organs and pelvic nodes). *	Presence of proven metastatic disease and/or in advanced disease prior to surgery; If SLN is considered, for groin. *	For tumors >2 cm, suspected LN involvement, thoracic and abdomen CT *; in suspected metastatic disease, thoraco–abdomen– pelvic CT.	For treatment planning *; in advanced stages for detecting metastases to pelvic and groin *	Evaluate groin nodes. *	NM.
**MRI**	Yes, to evaluate disease extension. *	*	In clinically invasive tumor (>T2), to evaluate extra-vulvar structures.	If SLN is considered, for groin *	For tumors > 2 cm, for suspected LN involvement. *	For treatment planning *; in advanced stages for detecting metastases to pelvis and groin. *	Evaluate groin nodes *; if stage > III, to evaluate local extension. *	NM.
**PET-CT**	Yes, to evaluate disease extension *	If N+ or if T3/T4.	Systemic staging. *	Not routinary, in presence of proven metastatic disease and/or in advanced disease prior to surgery.	NM.	For larger tumors to detect metastases, and in recurrence setting.	If stage >III, to evaluate disease extent. *	NM.
**FNA**	NM.	If enlarged inguinal nodes detected.	Yes, for suspected nodes—US-guided.	For suspected nodes.	NM.	NM.	If clinical or radiologic, suspected groin nodes.	Suspected groin nodes prior to RT.
**Cystoscopy and/or anoscopy**	Yes, if indicated.	NM.	NM.	NM.	NM.	NM.	Yes.	NM.
**HIV screening**	Yes, in younger patients.	Yes.	NM.	In prevention and screening setting.	NM.	Yes.	Yes.	NM.
**Others**	CBC, liver, and renal tests; counseling for smoking cessation; cervical HPV test.	CBC, screening for other HPV-associated tumors; FS counseling if appropriate.	HPV test; TSV/TR/TP US if center has expert sonographer.	NM.	NM.	CBC, liver profile, chest X-ray.	ASA score, Balducci score (onco-geriatric).	NM.

Legend: EUA: examination under anesthesia; LNI: lymph node invasion; CBC: complete blood count; NM: not mentioned; ND: not detailed; pts: patients; GV: gynecological visit; N: nodes, +: positive; T2/T3/T4: stages according to 8th edition of TNM staging tumor system; FS: fertility sparing; SLN: sentinel node biopsy, IF: inguinofemoral (nodes); TSV/TR/TP: transvaginal/transrectal/transperineal; US: ultrasound; ASA: anesthesiological score for patient global performance status; RT: radiotherapy. *: there is no criterion of preference or order in the use of imaging methods. (*): include an exam of inguinal, retro-crural, and supra-clavicular areas.

**Table 2:** Early-stage vulvar cancer treatment, groin assessment.

Staging Sistems	NCCN	ASCO	ESGO	IGCS	BGCS	FIGO	French	JSGO	RCOG
**Stage IA (FIGO 2021)** **or** **T1aN0M0** **(≤2 cm and stromal invasion ≤ 1 mm)**	≤1 mm invasion: SPV; if R0 FU; if R1 resectable: re-excision→if R0: FU or RT, if R1: RT. If R1 unresectable: RT.	RPV + surgical staging (if DOI < 1 mm, it is not required) SNLB (unifocal T1N0 < 4 cm) or i/bIFLD according to lateralization. If R1, re-excision or RT. If close margin: RT in case of PNI or LVI +. If SLN miMet: IF RT; if SLN maMet: IFLD + (CHT−RT if total 2LNs+.	Radical local excision with R0, including dVIN; multifocal invasive disease, consider radical excision of each lesion; considering vulvectomy (function sparing). If R1: re-excision if feasible. >T1a; VCL (medial border < 1 cm from midline): bIFLNs surgical evaluation. uT < 4 cm, no LNs suspicious: SLN; if SLN not found, IFLD. ≥4 cm and/or multifocal disease: bIFLD. If LL, iIFLD and if +, also contralateral IFLD. Consider preserving saphenous vein.	By consensus: for I or IIstages, tumor < 4 cm with—IFLD and R0; only FU.	Excision anatomy and function sparing with all R0. For posterior lesions, consider reconstructive surgery.	Radical wide excision including preinvasive disease with R0 (surgical margins of 2 cm). SLN: tumors < 4 cm, unifocally confined to the vulva, stromal invasion < 1 mm; negative groin nodes.	IA FIGO 2009: Superficial PV with R0; if lesion near to the urethra, 1 cm resection is feasible. If multifocal lesion, superficial RV.	Tumors < 2 cm, stromal invasion ≦ 1 mm: wide local excision; if >1 mm: LL: radical local excision+ iIFLD; if VCL: RV+ bIFLD.	FIGO 2009: tumors < 2 cm and DOI < 1 mm: wide local excision.
**Stage IB (FIGO 2021)** **or** **T1bN0M0** **(>2 cm or stromal invasion >1 mm)**	For LL (≥2 cm from vulvar midline): RPV+ iIFLNs (SNLB or iIFLD); for VCL: RPV + bIFLNs (SNLB or bIFLD). * **			By consensus: tumors >2 cm from midline, if + iIFLDN; perform controlateral. * Tumor <4 cm (distant 2 cm from midline), SLN unilaterally uptaking; perform controlateral IFLD. *^2^	For LL (>1 cm from midline): radical wide excision. Anterior vulvectomy for peri-clitoris tumors; posterior vulvectomy for the posterior lesions (anal sphincter sparing). Multifocal disease: wide local excisions; consider RV.		RV until urogenital fascia with R0 + SLNB if lesion <4 cm; unilateral SLN if LL (>1 cm from midline); bilateral SLN if VCL. IFLD if positive nodes. If R1 with up to 8 mm: re-excision; if R1 < 5 mm: RT if unresectable residual disease. *	FIGO 2009: tumors > 2 cm resectable: LL→ radical local excision+ bIFLD or RV+ bIFLD. In VCL: RV + bIFLD.	FIGO 2009: DOI > 1 mm; tumors > 2 cm + triple incision technique for groin nodes dissection. *
**Stage II**	* for selected cases of stage II.	NM.	T2 is already stage FIGO III.	By majority: for T2, * as above, *^2^ for T2.	Excision of distal urethra and vagina should be considered; if anus involved: CHT-RT or neoadiuvant CHT-RT followed by surgery (sparing of continence loss).		Same indications of French IB. *		As above. *

**Legend**: −: negative; +: positive; FU: follow-up; SPV: simple partial vulvectomy; LL: lateral lesion; RPV: radical partial vulvectomy; iIFLN: ipsilateral inguinofemoral lymph-nodes; SNLB: sentinel lymph node biopsy; iIFLD: ipsilateral inguinofemoral lymphadenectomy; VCL: vulvar central lesion; bIFLD: bilateral inguinofemoral lymphadenectomy; bIFLNs: bilateral inguinofemoral lymph nodes; R0: clear margins; R1: positive margins; PNI: perineural invasion; LVI: lymphovascular invasion; miMet: micrometastases; maMet: macrometastases; CHT: chemotherapy; RT: radiotherapy; LNs: lymph nodes; dVIN: differentiated vulvar intraephitelial neoplasia; uT: unifocal tumors; PV: partial vulvectomy; RV: radical vulvectomy; DOI: depth of invasion. *: treatment strategy is the same. **: assessment of primary tumor and nodal surgical pathology; adjuvant therapy based on Primary Tumor Risk Factors; NM: not mentioned.

**Table 3:** Advanced-stage vulvar cancer treatment.

NCCN	ASCO	ESGO	IGCS	BGCS	FIGO	French	JSGO	RCOG
Unresectable disease if nodes are −: IFLD. Positive LNs: RT + CHT covering tumor, IF, and pelvic nodes; if negative: RT+CHT covering tumor and selective IF nodes. If nodes suspected on imaging: FNA on enlarged nodes and RT + CHT to IFLNs both pelvic and tumor. Same indications for unresectable nodes.	CHT-RT; if residual disease: biopsy +/− surgery.	CHT-RT in a specialized gynecological RT center in unresectable disease. Avoid treatment breaks. In patients not eligible for these options: platinum-based NACT to be evaluated in MDT.	By consensus: 2D RT including cobalt technique; cisplatin as radiosensitizing agent; concomitant CHT-RT. By majority: RV + bIFLD + RT; BSC if poor geriatric score and/or PS. RT for pathological nodal involvement to groin and pelvis; resected tumor including vulva and pelvic nodes. Carboplatin as radiosensitizing.	Definitive CHT-RT enrolling in clinical trials.	FNA or biopsy on suspected nodes; if negative: RT to groin and pelvic nodes; if positive: adjuvant RT or CHT-RT to groin and pelvic nodes; if no suspicious nodes: IFLD. Primary CHT-RT if unfit for surgery.	If resectable tumor: total RV + IFLD; if fixed nodes: inguino-iliac neoadiuvant RT. If positive margins: re-excision vs. RT. If positive nodes: pelvic RT. If non-resectable tumor: neodiuvant RT +/− CHT. If complete regression: confirmatory biopsies. If negative, adjuvant RT. If partial regression and resectable tumor: radical deep excision + RT if clear margins < 5 mm; if unresectable tumor: RT.	Neoadjuvant CHT-RT +. Pelvic exenteration if no node metastases.	RT, definitive or neoadiuvant if surgery implies function loss. Wide radical local excision with a minimum of 15 mm of free margins.

**Legend.** MDT: multidisciplinary team; IFLD: inguinofemoral lymphadenectomy; CHT: chemotherapy; RT: radiotherapy; RV: radical vulvectomy; LNs: lymph nodes; RT: radiotherapy; bIFLD: bilateral inguinofemoral lymphadenectomy; IF: inguinofemoral; NACT: neoadiuvant chemotherapy; BSC: best supportive care.

**Table 4:** Follow-up strategies.

	NCCN	ASCO	ESGO	BGCS	FIGO	French	JSGO	RCOG
**Timing**	Every 3–6 months for 2 y, every 6–12 months from 3 to 5 y; then annually based on risk of recurrence.	Every 3–4 months for 2 y, then every 6 months.	Six–eight weeks after treatment; every 3–4 months for 2 y; biannual or annual from 3 to 5 y.	Lifelong FU for HPV-independent VSCC.	Every 3–6 months for 2 y, then every 6–12 until 5 y.	Every 3–4 months for 1 y, then every 6 months from 2 y.	Every 1–3 months for 2 y, every 6 months from 3 to 5 y; then annually.	Every 3 months for 1 year, 6 months for the 2 y, then yearly.
**VG**	Yes.	Yes.	Symptom review, complete physical exam of vulva, skin bridge and IF nodes.	Report new lesions and be seen urgently.	Review of symptoms or side effects.	Perineal and groin nodes complete exam.	Inspection and palpation.	NM.
**Cervical citology**	As indicated for detection of lower genital neoplasia.	Yes.	6–12 mo after primary treatment.	NM.	NM.	NM.	Yes.	NM.
**Blood exams**	Based on clinical (symptoms and exam) suspected recurrence. *	NM.	Only based on risk of recurrence, clinical findings, and/or side effects. *	NM.	SCC-Ag based on preoperative value used as serum tumor marker FU.	NM.	Tumor markers.	NM.
**Imaging**	* As above NCCN.	As clinically indicated. MRI and/or PET TC 3–4 mo after end CHT-RT for advanced tumors.	* As above ESGO.	NM.	NM.	CT abdomen–pelvis in locally advanced tumors and/or with nodal involvement.	CT.	NM.
**Other**	Education for symptoms of recurrence and vulvar dystrophy, periodic self-examinations, lifestyle, obesity, exercise, sexual health smoking cessation, nutrition counseling, and potential long-term and late effects of treatment.	Treatment of sequelae: discuss HPV vaccination for secondary prevention.	Long-term surveillance if based on ongoing vulvar disease or treatment- related side effects.	Those with no recurrence of HPV-dependent VSCC or uVIN could be discharged to patient-initiated follow-up, with access to rapid re-referral after five years.	NM.	NM.	Biopsy and chest X-ray.	Based on patient ability to self-assessment.

**Legend.** mo: months; y: years; IF: inguinofemoral; SCC-Ag: antigen for squamous cell carcinoma; US: ultrasound; CHT-RT: chemoradiation; VSCC: vulvar squamous cell carcinoma. * same indications; NM: not mentioned.

**Table 5:** Recurrence treatment.

	NCCN	ESGO	IGCS	BGCS	FIGO	JSGO	RCOG
**Work-up**	CT chest/abdomen/pelvis or neck and groin PET-TC; confirmatory biopsy both locally and at distance of suspected lesions.	Vulvar exam and biopsies of suspected lesions (if feasible); US/MRI and/or CT (or PET-TC) of thorax/abdomen/pelvis.	NM.	CT or PET-TC if appropriate of thorax/abdomen/ pelvis.	NM.	NM.	NM.
**Surgery**	LR→PRV or total RV +/− i/bIFLD for LVR; PE for CR; re-excision if positive margins.	LVR: in clinical negative IFLNs, surgical groin restaging; re-excision if positive margins; PE in selected cases; FLD or debulking of suspicious nodes in suspected NR; debulking of enlarged pelvic nodes prior to CHT-RT; in selected cases, PE.	Salvage surgery for LR without nodal involvement, comorbidities and treated with surgery at first diagnosis; by majority add also the specific: without available RT; salvage surgery in the same conditions and if irradiated patients.	For LR and IF NR: re-excision. SLNB: to be performed if the new focus of recurrence meets criteria for primary SLN.	Yes. **	LR resectable: local resection; for IR not previous nodes removed: IFLD.	For IR not pre- irradiated to groin; palliative surgery as preirradiated groin recurrence.
**RT**	RT for negative margins and nodes and for positive margins and negative nodes; RT+BRT: as first recurrence treatment.	As alternative for LVR excised with positive margins; for oligometastatic IF/pelvic disease: consider stereotactic RT.	Salvage surgery + RT in NR in patients with comorbidities and contraindication to platin treated initially only with surgery also with RT available.	Palliative if surgery not feasible. *	NM.	For LR resectable as alternative to surgery; for LR unresectable in non-preirradiated patients as primary or NA; in the same cohort also as palliative; adjuvant after IFLD for IR.	For IR not pre- irradiated to groin with or without surgery.
**CHT**	Could be associated as adjuvant in the previous conditions; for multiple nodes and distant recurrences.	If local therapies not feasible for NR; for distant recurrences.	Carboplatin if cisplatin ineligible as radiosensitizer; cisplatin after salvage surgery for NR; carboplatin/paclitaxel after salvage surgery for NR in pretreated with RT-CHT and surgery.	Palliative if surgery not feasible *; to be considered for distant metastases (not yet standardized!).	NA or palliative CHT.	In LR unresectable patients preirradiated; for IR, previous nodes removed and irradiated.	Considered but not detailed.
**RT-CHT**	RT+CHT: Surgical margins (−) with IFLNs (+); RT+BRT+CHT: margins (+) and IFLNs; RT+BRT+CHT: as first recurrence treatment in LR; RT+CHT: nodal and distance recurrences.	In RT naive for LVR; in NR following surgery; in pelvic nodal recurrence. consider radiosensitizer CHT agent in combined therapies.	NM.	Palliative if surgery not feasible. *	Yes. **	For LR unresectable as NA or primary; as adjuvant in IR after IFLD; for IR, previous nodes removed not irradiated.	
**BSC**	Distant recurrences and pelvic nodes.	If incurable.	NM.	NM.	Yes. **	Alternative for LR unresectable non-preirradiated; for IR, previous nodes removed and irradiated.	As palliation if groin recurrence and pretreated with surgery and RT.
**Immuno-** **therapy**	Pembrolizumab as 2L for PDL1-positive recurrent VCs.	NM.	NM.	NM.	A targeted agent if available.	NM.	NM.

**Legend.** PRV: partial radical vulvectomy; RV: radical vulvectomy; iIFLD: ipsilateral inguinofemoral lymphadenectomy; bIFLD: bilateral inguinofemoral lymphadenectomy; LVR: local vulvar recurrence; PE: pelvic exenteration; CR: central recurrence; BRT: brachytherapy; IFLNs: inguinofemoral lymph nodes; LR: local recurrence; 2L: second line; NR: nodal recurrence; IF: inguinofemoral; SLNB: sentinel node biopsy; NA: neoadiuvant; IR: inguinal recurrence. * choice depends on patient’s preferences and fitness status. ** FIGO 2021 guidelines only list possibilities for recurrence treatment; NM: not mentioned.

## Data Availability

No new data were created or analyzed in this study. Data sharing is not applicable to this article.
