# Correction: Restaino et al. Management of Patients with Vulvar Cancers: A Systematic Comparison of International Guidelines (NCCN–ASCO–ESGO–BGCS–IGCS–FIGO–French Guidelines–RCOG). *Cancers* 2025, *17*, 186

**DOI:** 10.3390/cancers17193117

**Published:** 2025-09-25

**Authors:** Stefano Restaino, Giulia Pellecchia, Martina Arcieri, Giorgio Bogani, Cristina Taliento, Pantaleo Greco, Lorenza Driul, Vito Chiantera, Rosa Pasqualina De Vincenzo, Giorgia Garganese, Francesco Sopracordevole, Violante Di Donato, Andrea Ciavattini, Paolo Scollo, Giovanni Scambia, Giuseppe Vizzielli, Gynecologic Oncology Group

**Affiliations:** 1 Clinic of Obstetrics and Gynecology, “Santa Maria della Misericordia” University Hospital, Azienda Sanitaria Universitaria Friuli Centrale, 33100 Udine, Italy; stefano.restaino@asufc.sanita.fvg.it (S.R.); pellecchia.giulia001@spes.uniud.it (G.P.); lorenza.driul@uniud.it (L.D.); giuseppe.vizzielli@uniud.it (G.V.); 2 PhD School in Biomedical Sciences, Gender Medicine, Child and Women Health, University of Sassari, 07100 Sassari, Italy; 3 Department of Medicine, University of Udine, 33100 Udine, Italy; 4 Gynaecological Oncology Unit, Fondazione IRCCS Istituto Nazionale dei Tumori di Milano, 20133 Milano, Italy; giorgio.bogani@istitutotumori.mi.it; 5 Department of Medical Sciences, Obstetrics and Gynecology Unit, University of Ferrara, 44121 Ferrara, Italy; cristina.taliento@unife.it (C.T.); grcptl@unife.it (P.G.); 6 Department of Health Promotion, Mother and Child Care, Internal Medicine and Medical Specialties (PROMISE), University of Palermo, 90127 Palermo, Italy; vito.chiantera@istitutotumori.na.it; 7 Unit of Gynecologic Oncology, National Cancer Institute, IRCCS, Fondazione “G. Pascale”, 80131 Naples, Italy; 8 Department of Women’s, Child and Public Health Sciences, UOC Gynecology Oncology, Fondazione Policlinico Universitario A. Gemelli IRCCS, 00168 Rome, Italy; rosapasqualina.devincenzo@policlinicogemelli.it (R.P.D.V.); giovanni.scambia@policlinicogemelli.it (G.S.); 9 Female External Genital Surgery Unit, Division of Gynecological Oncology, Department of Women’s, Children’s and Public Health Sciences, Fondazione Policlinico Universitario A. Gemelli IRCCS, 00168 Rome, Italy; giorgia.garganese@policlinicogemelli.it; 10 Gynecologic Oncology Unit, Centro di Riferimento Oncologico, National Cancer Institute, 33081 Aviano, Italy; fsopracordevole@cro.it; 11 Department of Gynecological, Obstetrical and Urological Sciences, “Sapienza” University of Rome, 00185 Rome, Italy; violante.didonato@uniroma1.it; 12 Woman’s Health Sciences Department, Gynecologic Section, Polytechnic University of Marche, 60121 Ancona, Italy; a.ciavattini@staff.univpm.it; 13 Department of Obstetrics and Gynecology, Cannizzaro Hospital, University of Enna “Kore” (D’Agate and Scibilia), 95126 Catania, Italy; pscollo@unict.it

There was an error in the original publication [1], regarding the cited reference [52]. The citation referred to “Tagliaferri, L.; Garganese, G.; D’Aviero, A.; Lancellotta, V.; Fragomeni, S.M.; Fionda, B.; Casà, C.; Gui, B.; Perotti, G.; Gentileschi, S.; et al. Multidisciplinary personalized approach in the management of vulvar cancer—The Vul.Can Team experience. *Int. J. Gynecol. Cancer* **2020**, *30*, 932–938”.

However, the results described in that paragraph more accurately correspond to the article by Maggino et al.: “Maggino, T.; Landoni, F.; Sartori, E.; Zola, P.; Gadducci, A.; Alessi, C.; Soldà, M.D.; Coscio, S.; Spinetti, G.; Maneo, A.; et al. Patterns of recurrence in patients with squamous cell carcinoma of the vulva: A multicenter CTF study. *Cancer* **2000**, *89*, 116–122.”

The original reference has now been replaced with the new one.

The authors apologize for any inconvenience caused and state that the scientific conclusions are unaffected. This correction was approved by the Academic Editor. The original publication has also been updated.
